# Criticality in cortical ensembles is supported by complex functional networks

**DOI:** 10.1186/1471-2202-15-S1-O15

**Published:** 2014-07-21

**Authors:** Paolo Massobrio, Valentina Pasquale, Sergio Martinoia

**Affiliations:** 1 Department of Informatics, Bioengineering, Robotics and Systems Engineering (DIBRIS), University of Genova, Genova, 16145, Italy; 2 Department of Neuroscience and Brain Technologies - NTECH, Istituto Italiano di Tecnologia (IIT), 16163, Genova, Italy

## 

Complex network topologies represent the necessary substrate to support complex brain function. It is widely recognized that the topological features of cortical networks are tightly linked to aspects of brain function by supporting which electrophysiological patterns can and cannot occur.

In this work, we investigated the interplay between network topology and spontaneous dynamics within the framework of neuronal avalanches and self-organized criticality (SOC) [1]. The main goal of this study is to sustain the hypothesis that the emergence of critical states, which in their turn would optimize functional properties in the cortex, is supported by specific complex network topologies. Experimental evidences showed that dissociated cortical assemblies coupled to Micro-Electrode Arrays (MEAs) can exhibit scale-free distributions of neuronal avalanches [2], a hallmark of SOC, thus demonstrating that they preserve self-organization properties featured by *in vivo*-formed cell assemblies [3]. However, the determinants of the emergence of different dynamical states (critical, subcritical or supercritical) remain unclear. Here, we adopted a reverse-engineering approach, by making use of an *in silico* neuronal network model reproducing the spiking and bursting activity of biological networks to explore the relationship between connectivity and dynamics. In our computational network model, connectivity is known *a priori* and thus it is possible to establish interdependencies between the avalanche distributions and the actual connectivity. Network topologies were designed following the canonical architectures of scale-free, random, and small-world graphs [4]. We simulated the spontaneous activity, by sweeping the most common parameters used to characterize these graphs, such as clustering coefficient, connection density, synaptic weight distributions, etc. [5]. From the simulations, we found that: (i) random networks only showed super-critical dynamics in a physiologically relevant domain of activity parameters (e.g. firing rate); (ii) scale-free and small-world architectures may account for the variability observed in the experimental data and the transition from subcriticality to criticality is ruled by the degree of “small-worldness”; (iii) excitation and inhibition should be appropriately balanced to allow for criticality [6].
